# 2,6-Di(pyrrolidin-1-yl)pyridinium chloride monohydrate

**DOI:** 10.1107/S160053681002427X

**Published:** 2010-06-26

**Authors:** Mohammad T. M. Al-Dajani, Hassan H. Abdallah, Nornisah Mohamed, Jia Hao Goh, Hoong-Kun Fun

**Affiliations:** a School of Pharmaceutical Sciences, Universiti Sains Malaysia, 11800 USM, Penang, Malaysia; b School of Chemical Sciences, Universiti Sains Malaysia, 11800 USM, Penang, Malaysia; c X-ray Crystallography Unit, School of Physics, Universiti Sains Malaysia, 11800 USM, Penang, Malaysia

## Abstract

In the organic cation of the title compound, C_13_H_20_N_3_^+^·Cl^−^·H_2_O, the two pyrrolidine rings adopt twisted conformations. The pyridine ring makes dihedral angles of 14.57 (6) and 23.96 (6)° with the mean planes of the pyrrolidine rings. In the crystal structure, pairs of bifurcated inter­molecular O—H⋯Cl hydrogen bonds link the water mol­ecules and chloride anions into an *R*_4_^4^(8) ring motif. Inter­molecular N—H⋯Cl, C—H⋯Cl and C—H⋯O hydrogen bonds further inter­connect these rings with the organic cations into a two-dimensional network parallel to the *bc* plane.

## Related literature

For general background to and applications of the title compound, see: Cornell *et al.* (2003 ▶); Fetzner (1998 ▶); Padoley *et al.* (2008 ▶); Xue & Warshawsky (2005 ▶); Zhu *et al.* (2003 ▶). For puckering analysis and ring conformations, see: Cremer & Pople (1975 ▶). For graph-set descriptions of hydrogen-bond ring motifs, see: Bernstein *et al.* (1995 ▶). For reference bond-length data, see: Allen *et al.* (1987 ▶). For related structures, see: Al-Dajani *et al.* (2009 ▶); Rubin-Preminger & Englert (2007 ▶). For the stability of the temperature controller used for the data collection, see: Cosier & Glazer (1986 ▶).
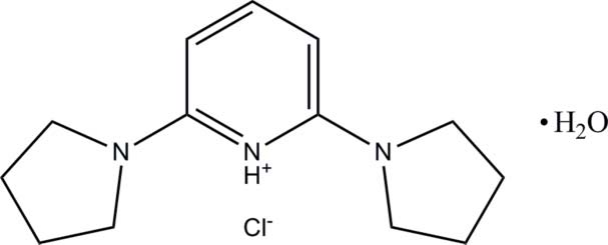


## Experimental

### 

#### Crystal data

C_13_H_20_N_3_^+^·Cl^−^·H_2_O
*M* *_r_* = 271.79
Monoclinic, 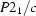

*a* = 11.5728 (15) Å
*b* = 12.2724 (16) Å
*c* = 11.3622 (16) Å
β = 119.214 (2)°
*V* = 1408.5 (3) Å^3^
*Z* = 4
Mo *K*α radiation
μ = 0.27 mm^−1^
*T* = 100 K
0.36 × 0.25 × 0.21 mm

#### Data collection

Bruker APEXII DUO CCD area-detector diffractometer
Absorption correction: multi-scan (*SADABS*; Bruker, 2009 ▶) *T*_min_ = 0.911, *T*_max_ = 0.947
20960 measured reflections
5073 independent reflections
4506 reflections with *I* > 2σ(*I*)
*R*_int_ = 0.028

#### Refinement

*R*[*F* ^2^ > 2σ(*F* ^2^)] = 0.033
*wR*(*F* ^2^) = 0.129
*S* = 1.26
5073 reflections
163 parameters
H-atom parameters constrained
Δρ_max_ = 0.85 e Å^−3^
Δρ_min_ = −0.47 e Å^−3^

### 

Data collection: *APEX2* (Bruker, 2009 ▶); cell refinement: *SAINT* (Bruker, 2009 ▶); data reduction: *SAINT*; program(s) used to solve structure: *SHELXTL* (Sheldrick, 2008 ▶); program(s) used to refine structure: *SHELXTL*; molecular graphics: *SHELXTL*; software used to prepare material for publication: *SHELXTL* and *PLATON* (Spek, 2009 ▶).

## Supplementary Material

Crystal structure: contains datablocks global, I. DOI: 10.1107/S160053681002427X/is2565sup1.cif

Structure factors: contains datablocks I. DOI: 10.1107/S160053681002427X/is2565Isup2.hkl

Additional supplementary materials:  crystallographic information; 3D view; checkCIF report

## Figures and Tables

**Table 1:** Hydrogen-bond geometry (Å, °)

*D*—H⋯*A*	*D*—H	H⋯*A*	*D*⋯*A*	*D*—H⋯*A*
N2—H1*N*2⋯Cl1	0.84	2.45	3.2246 (10)	153
O1*W*—H1*W*1⋯Cl1	0.91	2.35	3.2502 (11)	169
O1*W*—H2*W*1⋯Cl1^i^	0.82	2.45	3.2594 (11)	171
C1—H1*B*⋯Cl1	0.97	2.76	3.5100 (11)	135
C7—H7*A*⋯O1*W*^ii^	0.93	2.35	3.2122 (15)	154
C13—H13*A*⋯Cl1	0.97	2.78	3.5555 (13)	138

Symmetry codes: (i) 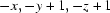; (ii) 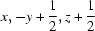.

**Fig. 1.**
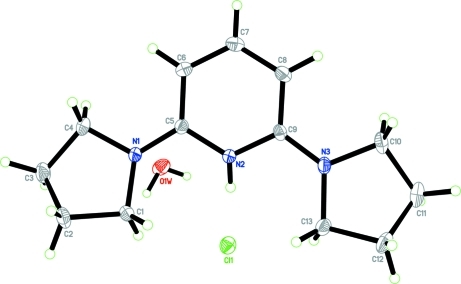
The molecular structure of the title salt, showing the atomic numbering scheme. Displacement ellipsoids are drawn at the 50% probability level.

**Fig. 2.**
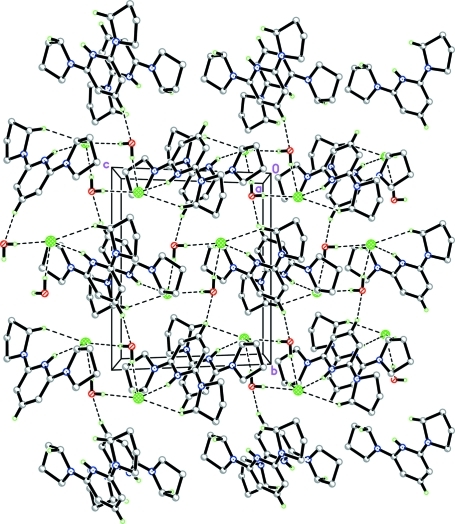
The crystal structure of the title salt, viewed along the a axis, showing a two-dimensional array parallel to the bc plane. H atoms not involved in intermolecular hydrogen bonds (dashed lines) have been omitted for clarity.

## supplementary crystallographic information

### Comment

Nitrogen heterocyclic compounds have received a lot of attention especially by the environment scientists. The main sources of these compounds in the environment are the coal gasification, shale oil extraction and pesticide production (Zhu *et al.*, 2003; Fetzner, 1998). The metabolic activation of the heterocyclic compounds and the DNA damage produced (Xue & Warshawsky, 2005) as well as their roles as a pollutants (Padoley *et al.*, 2008) and their deposition on land and coastal environments  (Cornell *et al.*, 2003) have been reported. The title compound can be used for the synthesis of new organometallic complexes and in the field of biological activity and drug design.

The asymmetric unit of the title salt comprises of a protonated 2,6-di(pyrrolidin-1-yl)pyridinium cation, a chloride anion and a water molecule (Fig. 1). In the organic cation, the two pyrrolidine rings adopts twisted conformations (Cremer & Pople, 1975). The puckering parameters are Q = 0.3969 (12) Å, φ = 94.02 (16)° for C1-C4/N1; and Q = 0.3732 (13) Å, φ = 274.73 (17)° for C10-C13/N3. The essentially planar pyridine ring (C5-C9/N2) makes dihedral angles of 23.96 (6) and 14.57 (6)°, respectively, with the mean planes formed through the C1-C4/N1 and C10-C13/N3 pyrrolidine rings. Comparing to the unprotonated structure (Rubin-Preminger & Englert, 2007), protonation at atom N2 has lead to a slight increase in the C5—N2—C9 angle to 122.97 (8)°. The bond lengths (Allen *et al.*, 1987) and angles are within normal ranges and comparable to a related pyridine structure (Al-Dajani *et al.*, 2009).

In the crystal structure (Fig. 2), the chloride anions provide the most extensive part as hydrogen bond acceptors. Pairs of intermolecular O1W—H1W1···Cl1 and O1W—H2W1···Cl1 bifurcated hydrogen bonds (Table 1) link the chloride anions and water molecules into *R*^4^_4_(8) ring motifs (Bernstein *et al.*, 1995) in a *DAAD* manner. These ring motifs are further interconnected with the organic cations into two-dimensional arrays parallel to the *bc* plane *via* intermolecular N2—H1N2···Cl1, C1—H1B···Cl1, C7—H7A···O1W and C13—H13A···Cl1 hydrogen bonds (Table 1).

### Experimental

In a two-neck round bottom flask, pyridine (0.01 mol, 1.0 g) was dissolved in THF (50 ml). The flask was connected to dropping funnel containing anhydrous aluminum chloride (2.7 g, 0.02 mol) dissolved in THF (25 ml) and ended with anhydrous calcium chloride drying tube. In an ice bath, the aluminum chloride solution was added in small portions and the temperature was maintained between 273–278 K during the addition. The mixture was refluxed for 30 mins at 323–328 K under dry condition. Pyrrolidine (0.02 mol, 1.5 g) was added in small portions to the formed red colour reaction mixture. After stirring for 1 h, the mixture was decanted on ice water and the organic layer was extracted with butanol. The solvent was evaporated by using the rotary evaporator. Deep brown single crystals were formed after one week at room temperature and washed with methanol and dried at room temperature.

### Refinement

H atoms bound to N and O atoms were located in a difference Fourier map (N—H = 0.84 and O—H = 0.82–0.91 Å) and constrained to ride with their parent atoms, with *U*_iso_(H) = 1.2*U*_eq_(N) or 1.5*U*_eq_(O). The remaining H atoms were placed in calculated positions (C—H = 0.93 or 0.97 Å), with *U*_iso_(H) = 1.2*U*_eq_(C).

### Crystal data

**Table:**

C_13_H_20_N_3_^+^·Cl^−^·H_2_O	*F*(000) = 584
*M**_r_* = 271.79	*D*_x_ = 1.282 Mg m^−^^3^
Monoclinic, *P*2_1_/*c*	Mo *K*α radiation, λ = 0.71073 Å
Hall symbol: -P 2ybc	Cell parameters from 9025 reflections
*a* = 11.5728 (15) Å	θ = 3.6–35.1°
*b* = 12.2724 (16) Å	µ = 0.27 mm^−^^1^
*c* = 11.3622 (16) Å	*T* = 100 K
β = 119.214 (2)°	Block, brown
*V* = 1408.5 (3) Å^3^	0.36 × 0.25 × 0.21 mm
*Z* = 4	

### Data collection

**Table:**

Bruker APEXII DUO CCD area-detector diffractometer	5073 independent reflections
Radiation source: fine-focus sealed tube	4506 reflections with *I* > 2σ(*I*)
graphite	*R*_int_ = 0.028
φ and ω scans	θ_max_ = 32.5°, θ_min_ = 3.6°
Absorption correction: multi-scan (*SADABS*; Bruker, 2009)	*h* = −17→17
*T*_min_ = 0.911, *T*_max_ = 0.947	*k* = −18→18
20960 measured reflections	*l* = −17→17

### Refinement

**Table:**

Refinement on *F*^2^	Primary atom site location: structure-invariant direct methods
Least-squares matrix: full	Secondary atom site location: difference Fourier map
*R*[*F*^2^ > 2σ(*F*^2^)] = 0.033	Hydrogen site location: inferred from neighbouring sites
*wR*(*F*^2^) = 0.129	H-atom parameters constrained
*S* = 1.26	*w* = 1/[σ^2^(*F*_o_^2^) + (0.0709*P*)^2^ + 0.155*P*] where *P* = (*F*_o_^2^ + 2*F*_c_^2^)/3
5073 reflections	(Δ/σ)_max_ = 0.001
163 parameters	Δρ_max_ = 0.85 e Å^−^^3^
0 restraints	Δρ_min_ = −0.47 e Å^−^^3^

### Special details

**Table:**

Experimental. The crystal was placed in the cold stream of an Oxford Cryosystems Cobra open-flow nitrogen cryostat (Cosier & Glazer, 1986) operating at 100.0 (1)K.
Geometry. All esds (except the esd in the dihedral angle between two l.s. planes) are estimated using the full covariance matrix. The cell esds are taken into account individually in the estimation of esds in distances, angles and torsion angles; correlations between esds in cell parameters are only used when they are defined by crystal symmetry. An approximate (isotropic) treatment of cell esds is used for estimating esds involving l.s. planes.
Refinement. Refinement of F^2^ against ALL reflections. The weighted R-factor wR and goodness of fit S are based on F^2^, conventional R-factors R are based on F, with F set to zero for negative F^2^. The threshold expression of F^2^ > 2sigma(F^2^) is used only for calculating R-factors(gt) etc. and is not relevant to the choice of reflections for refinement. R-factors based on F^2^ are statistically about twice as large as those based on F, and R- factors based on ALL data will be even larger.

### Fractional atomic coordinates and isotropic or equivalent isotropic displacement parameters (Å^2^)

**Table:**

	*x*	*y*	*z*	*U*_iso_*/*U*_eq_	
Cl1	0.19322 (2)	0.62909 (2)	0.66837 (2)	0.01949 (8)	
N1	0.36262 (7)	0.57142 (7)	1.04747 (8)	0.01531 (15)	
N2	0.14198 (7)	0.52984 (6)	0.90169 (8)	0.01275 (14)	
H1N2	0.1409	0.5734	0.8446	0.015*	
N3	−0.08202 (7)	0.50501 (7)	0.75519 (8)	0.01540 (15)	
C1	0.34608 (9)	0.68840 (8)	1.01429 (10)	0.01729 (17)	
H1A	0.2653	0.7163	1.0090	0.021*	
H1B	0.3446	0.7022	0.9295	0.021*	
C2	0.46810 (9)	0.73903 (9)	1.13225 (11)	0.0226 (2)	
H2A	0.4514	0.7568	1.2058	0.027*	
H2B	0.4954	0.8045	1.1045	0.027*	
C3	0.57226 (10)	0.64951 (9)	1.17295 (12)	0.0231 (2)	
H3A	0.6087	0.6468	1.1121	0.028*	
H3B	0.6437	0.6601	1.2644	0.028*	
C4	0.49368 (9)	0.54686 (9)	1.16178 (10)	0.02016 (19)	
H4A	0.5325	0.4835	1.1435	0.024*	
H4B	0.4888	0.5344	1.2436	0.024*	
C5	0.26136 (9)	0.50134 (7)	1.01015 (9)	0.01296 (16)	
C6	0.27123 (9)	0.40293 (8)	1.07522 (10)	0.01685 (17)	
H6A	0.3516	0.3802	1.1467	0.020*	
C7	0.15781 (10)	0.33922 (8)	1.03070 (10)	0.01769 (18)	
H7A	0.1637	0.2735	1.0740	0.021*	
C8	0.03737 (10)	0.36996 (7)	0.92503 (10)	0.01668 (18)	
H8A	−0.0372	0.3266	0.8984	0.020*	
C9	0.02951 (8)	0.46816 (7)	0.85822 (9)	0.01324 (16)	
C10	−0.20146 (9)	0.43731 (9)	0.69091 (11)	0.02049 (19)	
H10A	−0.2389	0.4269	0.7501	0.025*	
H10B	−0.1827	0.3667	0.6657	0.025*	
C11	−0.29411 (10)	0.50344 (10)	0.56689 (11)	0.0251 (2)	
H11A	−0.3857	0.4926	0.5440	0.030*	
H11B	−0.2836	0.4836	0.4900	0.030*	
C12	−0.25148 (10)	0.62099 (9)	0.60917 (11)	0.0217 (2)	
H12A	−0.2777	0.6675	0.5312	0.026*	
H12B	−0.2893	0.6492	0.6627	0.026*	
C13	−0.10123 (9)	0.61292 (8)	0.69258 (10)	0.01769 (18)	
H13A	−0.0603	0.6171	0.6359	0.021*	
H13B	−0.0654	0.6700	0.7601	0.021*	
O1W	0.07681 (10)	0.38244 (7)	0.62242 (9)	0.02792 (19)	
H1W1	0.0986	0.4542	0.6261	0.042*	
H2W1	0.0048	0.3828	0.5536	0.042*	

### Atomic displacement parameters (Å^2^)

**Table:**

	*U*^11^	*U*^22^	*U*^33^	*U*^12^	*U*^13^	*U*^23^
Cl1	0.01614 (12)	0.02374 (14)	0.01981 (13)	0.00041 (7)	0.00973 (10)	0.00133 (8)
N1	0.0105 (3)	0.0137 (3)	0.0163 (3)	−0.0004 (2)	0.0023 (3)	0.0012 (3)
N2	0.0111 (3)	0.0121 (3)	0.0131 (3)	−0.0003 (2)	0.0044 (3)	0.0008 (2)
N3	0.0101 (3)	0.0150 (3)	0.0172 (3)	−0.0008 (2)	0.0036 (3)	−0.0021 (3)
C1	0.0140 (4)	0.0132 (4)	0.0203 (4)	−0.0011 (3)	0.0050 (3)	−0.0005 (3)
C2	0.0140 (4)	0.0192 (4)	0.0281 (5)	−0.0034 (3)	0.0054 (4)	−0.0073 (4)
C3	0.0116 (4)	0.0236 (5)	0.0275 (5)	−0.0019 (3)	0.0045 (4)	−0.0028 (4)
C4	0.0111 (4)	0.0231 (5)	0.0189 (4)	0.0012 (3)	0.0016 (3)	0.0022 (3)
C5	0.0115 (3)	0.0134 (4)	0.0129 (4)	0.0006 (3)	0.0051 (3)	−0.0003 (3)
C6	0.0167 (4)	0.0154 (4)	0.0165 (4)	0.0017 (3)	0.0066 (3)	0.0032 (3)
C7	0.0207 (4)	0.0133 (4)	0.0207 (4)	0.0007 (3)	0.0114 (3)	0.0023 (3)
C8	0.0171 (4)	0.0133 (4)	0.0208 (4)	−0.0020 (3)	0.0101 (3)	−0.0009 (3)
C9	0.0116 (3)	0.0128 (4)	0.0150 (4)	−0.0011 (3)	0.0063 (3)	−0.0031 (3)
C10	0.0126 (4)	0.0214 (4)	0.0242 (5)	−0.0043 (3)	0.0063 (3)	−0.0079 (4)
C11	0.0119 (4)	0.0369 (6)	0.0209 (5)	−0.0010 (4)	0.0037 (3)	−0.0053 (4)
C12	0.0125 (4)	0.0310 (5)	0.0200 (5)	0.0052 (3)	0.0066 (3)	0.0054 (4)
C13	0.0124 (4)	0.0204 (4)	0.0189 (4)	0.0022 (3)	0.0066 (3)	0.0034 (3)
O1W	0.0333 (4)	0.0220 (4)	0.0218 (4)	0.0079 (3)	0.0082 (3)	−0.0016 (3)

### Geometric parameters (Å, °)

**Table:**

N1—C5	1.3444 (11)	C5—C6	1.3914 (13)
N1—C4	1.4681 (12)	C6—C7	1.3935 (14)
N1—C1	1.4729 (13)	C6—H6A	0.9300
N2—C9	1.3724 (11)	C7—C8	1.3759 (14)
N2—C5	1.3740 (11)	C7—H7A	0.9300
N2—H1N2	0.8360	C8—C9	1.4034 (13)
N3—C9	1.3289 (11)	C8—H8A	0.9300
N3—C10	1.4659 (12)	C10—C11	1.5229 (16)
N3—C13	1.4680 (13)	C10—H10A	0.9700
C1—C2	1.5261 (13)	C10—H10B	0.9700
C1—H1A	0.9700	C11—C12	1.5244 (17)
C1—H1B	0.9700	C11—H11A	0.9700
C2—C3	1.5263 (15)	C11—H11B	0.9700
C2—H2A	0.9700	C12—C13	1.5241 (14)
C2—H2B	0.9700	C12—H12A	0.9700
C3—C4	1.5223 (15)	C12—H12B	0.9700
C3—H3A	0.9700	C13—H13A	0.9700
C3—H3B	0.9700	C13—H13B	0.9700
C4—H4A	0.9700	O1W—H1W1	0.9117
C4—H4B	0.9700	O1W—H2W1	0.8189
			
C5—N1—C4	120.85 (8)	C5—C6—H6A	120.8
C5—N1—C1	123.94 (7)	C7—C6—H6A	120.8
C4—N1—C1	112.06 (7)	C8—C7—C6	122.44 (9)
C9—N2—C5	122.97 (8)	C8—C7—H7A	118.8
C9—N2—H1N2	114.9	C6—C7—H7A	118.8
C5—N2—H1N2	119.1	C7—C8—C9	118.56 (9)
C9—N3—C10	121.35 (8)	C7—C8—H8A	120.7
C9—N3—C13	125.88 (8)	C9—C8—H8A	120.7
C10—N3—C13	112.77 (8)	N3—C9—N2	118.17 (8)
N1—C1—C2	102.89 (8)	N3—C9—C8	123.19 (8)
N1—C1—H1A	111.2	N2—C9—C8	118.64 (8)
C2—C1—H1A	111.2	N3—C10—C11	103.05 (9)
N1—C1—H1B	111.2	N3—C10—H10A	111.2
C2—C1—H1B	111.2	C11—C10—H10A	111.2
H1A—C1—H1B	109.1	N3—C10—H10B	111.2
C1—C2—C3	103.21 (8)	C11—C10—H10B	111.2
C1—C2—H2A	111.1	H10A—C10—H10B	109.1
C3—C2—H2A	111.1	C10—C11—C12	103.85 (8)
C1—C2—H2B	111.1	C10—C11—H11A	111.0
C3—C2—H2B	111.1	C12—C11—H11A	111.0
H2A—C2—H2B	109.1	C10—C11—H11B	111.0
C4—C3—C2	102.66 (8)	C12—C11—H11B	111.0
C4—C3—H3A	111.2	H11A—C11—H11B	109.0
C2—C3—H3A	111.2	C13—C12—C11	103.30 (8)
C4—C3—H3B	111.2	C13—C12—H12A	111.1
C2—C3—H3B	111.2	C11—C12—H12A	111.1
H3A—C3—H3B	109.1	C13—C12—H12B	111.1
N1—C4—C3	102.77 (8)	C11—C12—H12B	111.1
N1—C4—H4A	111.2	H12A—C12—H12B	109.1
C3—C4—H4A	111.2	N3—C13—C12	102.53 (8)
N1—C4—H4B	111.2	N3—C13—H13A	111.3
C3—C4—H4B	111.2	C12—C13—H13A	111.3
H4A—C4—H4B	109.1	N3—C13—H13B	111.3
N1—C5—N2	117.40 (8)	C12—C13—H13B	111.3
N1—C5—C6	123.70 (8)	H13A—C13—H13B	109.2
N2—C5—C6	118.90 (8)	H1W1—O1W—H2W1	99.6
C5—C6—C7	118.41 (9)		
			
C5—N1—C1—C2	150.43 (9)	C6—C7—C8—C9	1.27 (15)
C4—N1—C1—C2	−9.59 (11)	C10—N3—C9—N2	171.36 (8)
N1—C1—C2—C3	30.83 (11)	C13—N3—C9—N2	−8.05 (14)
C1—C2—C3—C4	−40.78 (11)	C10—N3—C9—C8	−9.15 (14)
C5—N1—C4—C3	−176.31 (9)	C13—N3—C9—C8	171.44 (9)
C1—N1—C4—C3	−15.59 (11)	C5—N2—C9—N3	177.66 (8)
C2—C3—C4—N1	34.27 (11)	C5—N2—C9—C8	−1.85 (13)
C4—N1—C5—N2	−179.62 (8)	C7—C8—C9—N3	−179.98 (9)
C1—N1—C5—N2	22.02 (13)	C7—C8—C9—N2	−0.49 (14)
C4—N1—C5—C6	0.56 (14)	C9—N3—C10—C11	−170.91 (9)
C1—N1—C5—C6	−157.79 (9)	C13—N3—C10—C11	8.57 (11)
C9—N2—C5—N1	−176.46 (8)	N3—C10—C11—C12	−28.80 (10)
C9—N2—C5—C6	3.37 (13)	C10—C11—C12—C13	38.50 (11)
N1—C5—C6—C7	177.33 (9)	C9—N3—C13—C12	−165.45 (9)
N2—C5—C6—C7	−2.48 (14)	C10—N3—C13—C12	15.10 (11)
C5—C6—C7—C8	0.23 (15)	C11—C12—C13—N3	−32.44 (10)

### Hydrogen-bond geometry (Å, °)

**Table:**

*D*—H···*A*	*D*—H	H···*A*	*D*···*A*	*D*—H···*A*
N2—H1N2···Cl1	0.84	2.45	3.2246 (10)	153
O1W—H1W1···Cl1	0.91	2.35	3.2502 (11)	169
O1W—H2W1···Cl1^i^	0.82	2.45	3.2594 (11)	171
C1—H1B···Cl1	0.97	2.76	3.5100 (11)	135
C7—H7A···O1W^ii^	0.93	2.35	3.2122 (15)	154
C13—H13A···Cl1	0.97	2.78	3.5555 (13)	138

Symmetry codes: (i) −*x*, −*y*+1, −*z*+1; (ii) *x*, −*y*+1/2, *z*+1/2.
